# Complete sequence and comparative analysis of the mitochondrial genome of the rare and endangered *Clematis acerifolia*, the first *clematis* mitogenome to provide new insights into the phylogenetic evolutionary status of the genus

**DOI:** 10.3389/fgene.2022.1050040

**Published:** 2023-01-04

**Authors:** Dan Liu, Kai Qu, Yangchen Yuan, Zhiheng Zhao, Ying Chen, Biao Han, Wei Li, Yousry A. El-Kassaby, Yangyang Yin, Xiaoman Xie, Boqiang Tong, Hongshan Liu

**Affiliations:** ^1^ Shandong Provincial Center of Forest and Grass Germplasm Resources, Jinan, China; ^2^ State Key Laboratory of Tree Genetics and Breeding, National Engineering Research Center of Tree Breeding and Ecological Restoration, College of Biological Sciences and Technology, Beijing Forestry University, Beijing, China; ^3^ College of Landscape Architecture and Tourism, Hebei Agricultural University, Baoding, China; ^4^ Guangxi Forestry Research Institute, Guangxi Key Laboratory of Special Non-wood Forest Cultivation &; Utilization, Nanning, China; ^5^ Forestry Protection and Development Service Center of Shandong Province, Jinan, China; ^6^ Department of Forest and Conservation Sciences, The University of British Columbia, Vancouver, BC, Canada; ^7^ Wuhan Benagen Technology Co., Ltd, Wuhan, China; ^8^ Hebei Hongya Mountain State-Owned Forest Farm, Baoding, China

**Keywords:** clematis, mitochondrial genome, organelle genome, Clematis acerifolia, phylogenetic relationship, repeats

## Abstract

*Clematis* is one of the large worldwide genera of the Ranunculaceae Juss. Family, with high ornamental and medicinal value. China is the modern distribution centre of *Clematis* with abundant natural populations. Due to the complexity and high morphological diversity of *Clematis*, the genus is difficult to classify systematically, and in particular, the phylogenetic position of the endangered *Clematis acerifolia* is highly controversial. The use of the mitochondrial complete genome is a powerful molecular method that is frequently used for inferring plants phylogenies. However, studies on *Clematis* mitogenome are rare, thus limiting our full understanding of its phylogeny and genome evolution. Here, we sequenced and annotated the *C. acerifolia* mt genome using Illumina short- and Nanopore long-reads, characterized the species first complete mitogenome, and performed a comparative phylogenetic analysis with its close relatives. The total length of the *C. acerifolia* mitogenome is 698,247 bp and the main structure is multi-branched (linear molecule 1 and circular molecule 2). We annotated 55 genes, including 35 protein-coding, 17 tRNA, and 3 rRNA genes. The *C. acerifolia* mitogenome has extremely unconserved structurally, with extensive sequence transfer between the chloroplast and mitochondrial organelles, sequence repeats, and RNA editing. The phylogenetic position of *C. acerifolia* was determined by constructing the species mitogenome with 24 angiosperms. Further, our *C. acerifolia* mitogenome characteristics investigation included GC contents, codon usage, repeats and synteny analysis. Overall, our results are expected to provide fundamental information for *C. acerifolia* mitogenome evolution and confirm the validity of mitochondrial analysis in determining the phylogenetic positioning of *Clematis* plants.

## Introduction

*Clematis acerifolia* Maxim of the *Clematis* genus in the Ranunculaceae family is a perennial shrub endemic to Beijing area and Taihang Mountains, China (Editorial Committee of flora of China, 1980). The species is characterized by its unique flowering (early spring flowering with large beautiful flowers) and habitat (growing on cliff faces, hence the name cliff flower). The species has narrow distribution with sparse populations and is threatened by *Parthenocissus quinquefolia* infestation and a range of human influences (Pang et al., 2022), that made it endangered and listed as a Grade II protected wild plant in 2021 (National Forestry and Grassland Administration (NFGA), 2021). The species unique flowing characteristics and its rare occurrence increased its ornamental and scientific values (Yuan et al., 2021). *C. acerifolia* was discovered by Karl Maximovich in the late 19th century (Maximowicz, 1879); however, the species suffers from limited research efforts. As the national germplasm survey continued to advance and the ornamental value of *C. acerifolia* continued to be explored, the species has gradually gained scholars attention with a series of morphological classification (Wang and Li, 2005), community characteristics (Yuan et al., 2021), population genetics (Lopez-Pujol et al., 2003), and ecological niches (Pang et al., 2022) studies.

*Clematis* is one of the world’s great genera, with over 350 known species, widely distributed on all continents except Antarctica and in the five major temperature zones, with greatest diversity in warm temperate and mountainous regions (Tamura, 1995). *Clematis* has been cultivated since the 16th century and more than 3, 500 horticultural cultivars have been named (Tamura, 1987; Grey-Wilson, 2000; Johnson, 2001; Lehtonen et al., 2016), occupying an important place in Japanese and Western gardens due to its high ornamental value (Christenhusz, 2000), while in China the species is widely known for its medicinal value (Zhang et al., 2018). However, the *Clematis* genera classification has been notoriously difficult, as its species are morphologically variable due to the genus complexity as well as the different characteristics emphasized by each classification system (Xie et al., 2011). In 1879, Maximovich published the original description of *C. acerifolia* taxonomy (Maximowicz, 1879). Based on the species flowering and leaf clusters traits, Volume 28 of the Flora of China classified *C. acerifolia* into Sect. *Cheiropsis* as a single species within Subsect. *Acerifoliae*; however, other morphological traits were different from those of Sect. *Cheiropsis* (Editorial Committee of flora of China, 1980; Yan et al., 2016). Geographically, plants of Sect. *Cheiropsis* are distributed in China, occurring in the south-western to central-western parts, whereas the range of *C. acerifolia* is far from Sect. *Cheiropsis* species, suggesting that *C. acerifolia* may not be closely related to Sect. *Cheiropsis* (Mu and Xie, 2011). Thus, the phylogenetic position of *C. acerifolia* remains to be highly controversial.

The chloroplast and nuclear genomes of *Clematis* are well sequenced and provided valuable information for their phylogeny (Yan et al., 2016; Xiang et al., 2019; Wang et al., 2021), evolution (Sheng et al., 2014), and conservation (Choi et al., 2021). However, it has been reported that the chloroplast genomes provided insufficient phylogenetic information to distinguish between closely related sugarcane cultivars, due to the cultivars’ recent origin and the genome conserved sequences (Evans et al., 2019). In contrast, the mitochondrial genomes (mitogenome) of plants are larger (Sloan et al., 2012), more plastic (Turnel et al., 2003; Rubinstein et al., 2013; Huang et al., 2022), and may contain increased phylogenetic signals (Duminil and Besnard, 2021). For instance, one of the main advantages of the mitogenome as a phylogenetic marker stem from the fact that its 36 genes have different rates of substitution, with some having higher rates than others, allowing relatively recent divisions to be tracked, and those having slower rates of substitution, deemed useful for elucidating deeper relationships (Saccone et al., 1999; Cho et al., 2004). In essence, mitogenomes can provide solutions for a very wide range of phylogenetic depths, from the timing of shallow divergence between populations of individual species to deep divergence within entire clades (Feng et al., 2021). The mitogenome has often been shown to help elucidate previously intractable phylogenies, clarifying the relationships between groups of phylogenetic difficulties that rapid radiation invalidates other markers. Other advantages of the mitogenome include: the wealth of genetic information it contains; its short binding time due to its haploid and matrilineal inheritance (very few species are biparental) (Moore, 1995; Breton et al., 2007); its relatively low recombination rate (Dong et al., 2018); and the clear homology of its coding genes (Stern and Palmer, 1984). In addition to sequence information, the mitogenome gene sequence (which will be discuss later in more details) is sometimes used as corroborating evidence in phylogenetics.

To date, more than 9,500 plant organelle genomes have been submitted to the NCBI’s (National Center for Biotechnology Information) organelle genome resource, but despite this, mitogenomes have rarely been studied in *Clematis*, with only 499 complete plant mitogenomes, less than one-tenth of those of chloroplasts, which is closely related to the complex structure of mitogenomes and the difficulty of their assembly. The development of genomics and sequencing technologies has provided new opportunities to address this issue, and third-generation sequencing allows access to large reads (Kumar et al., 2019), facilitating the assembly and annotation of plant organelle genomes. Xiang et al. (2019) completed the assembly of three chloroplast genomes of *C. acerifolia*, *Clematis smilacifolia* and *Clematis uncinata*, which included only minimal information on the organelle genome. The chloroplast genomes of the native Korean *Clematis brachyura* and *Clematis trichotoma* were later analyzed in more depth by Choi et al. (2021) as well. However, the evolution of mitochondria in *Clematis* remains unanswered. Deciphering this last unknown genetic material (mitogenome) is crucial to understanding the evolution and genomic resources of *Clematis*. Unfortunately, very little information is known about the genome of *Clematis*, and the mitogenome of *Clematis* has never been reported, which seriously hampers a comprehensive understanding of its genomic evolution.

In this study we characterized the first mitogenome of *Clematis*, and the complete mitogenome of *C. acerifolia* was sequenced and assembled using Nanopore and Illumina. The aims of this study were to: 1) determine the molecular characteristics of *C. acerifolia* mitogenome, 2) contribute to the understanding of the evolution of the *C. acerifolia* organelle genome through computational analysis of its GC content, codon usage preference, repeats, intracellular gene transfer analysis, RNA editing site prediction, and synteny analysis, 3) explore the evolutionary relationships between the *Clematis* species based on the protein-coding genes (PCGs) of the mitogenome, compared with the genomes of other angiosperms, and 4) synteny analysis was performed on closely related species of *Clematis* to investigate the effectiveness of mitogenome analysis in determining the phylogenetic position of *clematis* plants.

## Materials and methods

### Plant materials and sequencing

In May 2022, leaves of well-grown living *C. acerifolia* from 8-year-old plants were collected from the Hongyashan State-owned Forest Farm, Yi County, Baoding City, Hebei Province (N39°20′50″, E115°30′39″), and the collected young fresh leaves were stored in liquid nitrogen for reserve. Total plant genomic DNA and the remaining portion of the plant specimen (barcode number sdf1003248) were stored at the Shandong Forest and Grass Germplasm Resource Centre (code qytxl2022hys09), and total DNA was obtained following the steps of the blood/cell/tissue genomic DNA extraction kit (TIANamp Genoic DNA Kit) from Tiangen.

We used both the Nanopore GridION sequencing platform (Oxford Nanopore Technology, Oxford Science Park) and the Illumina Novaseq 6000 platform for sequencing and library construction, and obtained raw sequence data. Clean data were obtained by using Trimmomatic (Bolger et al., 2014). Here, we removed low-quality sequences, including sequences with a quality value of Q < 19 that accounted for more than 50% of the total bases and sequences in which more than 5% bases were "N."

### Genome assembly and annotation

We used a hybrid Illumina and Nanopore strategy to assemble the *C. acerifolia* mitogenome. Plant mitochondrial assemblies were first performed using the default parameters of GetOrganelle software (default parameters: v1.7.5) on second-generation DNA sequencing data to obtain a graphical representation of the mitochondrial genome (Jin et al., 2020). The *C. acerifolia* mitogenome was then visualized using Bandage software and single stretches of the chloroplast and nuclear genomes were manually removed (Wick et al., 2015). Finally, the Nanopore data was compared to the graphical mitogenome fragments using BWA software and the resulting Nanopore data was used to resolve the repetitive sequence regions of the graphical plant mitogenome (Li and Durbin, 2009). The multi-branched *C. acerifolia* mitogenome was finally obtained by the above method. We used Geseq to annotate the mitogenome of *C. acerifolia* (Tillich et al., 2017), and *Arabidopsis thaliana* (NC_037304.1) and *Aconitum kusnezoffii* (NC_053920.1) were selected as the reference genome for the protein-coding genes (PCGs) of the mitogenome. The tRNA and rRNA of the mitogenome were annotated using tRNAscan-SE and BLASTN (Lowe and Eddy, 1997; Chen et al., 2015), respectively. When errors were made in the annotation of the mitogenome, Apollo was used to manually correct the errors (Lewis et al., 2002). The annotation of the mitogenome of *C. acerifolia* has been uploaded to GenBank (Acession number: ON674117 and ON674118) and Figshare (doi: https://doi.org/10.6084/m9.figshare.21299757.v1).

### Codon preference and repetitive sequence analysis of the mitogenome of *C. acerifolia*

The protein-coding sequences of the genome were extracted using Phylosuite (Zhang et al., 2020). The PCGs of the mitogenome were analyzed for codon preference using Mega 7.0 and RSCU values were calculated (Kumar et al., 2016). The neutral plot analysis and ENC-plot analysis were used to perform codon preference analysis. The neutral plot analysis is based on the codon 3 GC content (GC3) as the horizontal coordinate and the average of codon 1 and 2 GC content (GC12) as the vertical coordinate, the correlation between GC12 and GC3 can determine the influencing factors of codon preference. When GC12 is significantly correlated with GC3, codon preference is influenced by mutation, and *vice versa* by natural selection; ENC-plot analysis was performed by establishing a coordinate system with GC3 as the horizontal coordinate and ENC as the vertical coordinate, and then adding the ENC standard curve to the coordinate system (Wang et al., 2022). If the gene points are distributed along or close to the standard curve, the codon preference of the gene is affected by mutation only, and conversely, if the gene points are far below the standard curve, the codon preference of the gene is affected by selection.

We used (MISA https://webblast.ipk-gatersleben.de/misa/) (Beier et al., 2017), TRF (https://tandem.bu.edu/trf/trf.unix.help.html) and REPuter web server (https://bibiserv.cebitec.uni-bielefeld.de/reputer/) to identify repetitive sequences including microsatellite sequence repeats, tandem repeats and scattered repeats (Benson, 1999; Stefan et al., 2001). The results were visualized using the Circos package (Zhang et al., 2013). In addition, we generated the edited mRNA sequences of PCGs manually, and then calculated the codon usage again. Codon usage before and after RNA editing was used for comparison.

### Chloroplast to mitochondrion DNA transformation and RNA editing prediction

The chloroplast genome was assembled and annotated using GetOrganelle and CPGAVAS2 (Shi et al., 2019; Jin et al., 2020), respectively. Homologous fragments were analyzed using BLASTN (Chen et al., 2015), and the results were visualized using the Circos package (Zhang et al., 2013). Prediction of RNA editing events based on the online website PREP suit (http://prep.unl.edu/) (Mower, 2009).

### Synteny and phylogenetic analysis

Based on sequence similarity, the *C. acerifolia* mitogenome was mapped with multiple synteny of closely related species using MCscanX (Wang et al., 2012). Pairwise comparisons of dot plots were generated and conserved co-linear blocks were plotted (Katoh et al., 2019). Mauve 2.3.1 was used for further comparative analysis of the collinearity of the mitogenomes of two closely related species (*Anemone maxima* and *A. kusnezoffii*) (Darling et al., 2004). Mitogenomes of closely related species were selected and downloaded (https://www.ncbi.nlm.nih.gov/) based on affinity, and then shared genes were extracted using PhyloSuite (Zhang et al., 2020), multiple sequence alignment analysis was performed using MAFFT with a bootstrap value of 1,000 (Katoh and Standley, 2013), and phylogenetic analysis using MRBAYES (Huelsenbeck and Ronquist, 2001). The results of the phylogenetic analysis were visualized in ITOL software (Letunic and Bork, 2019).

## Results

### *C. acerifolia* mitochondrial genomes characterization

**FIGURE 1:**
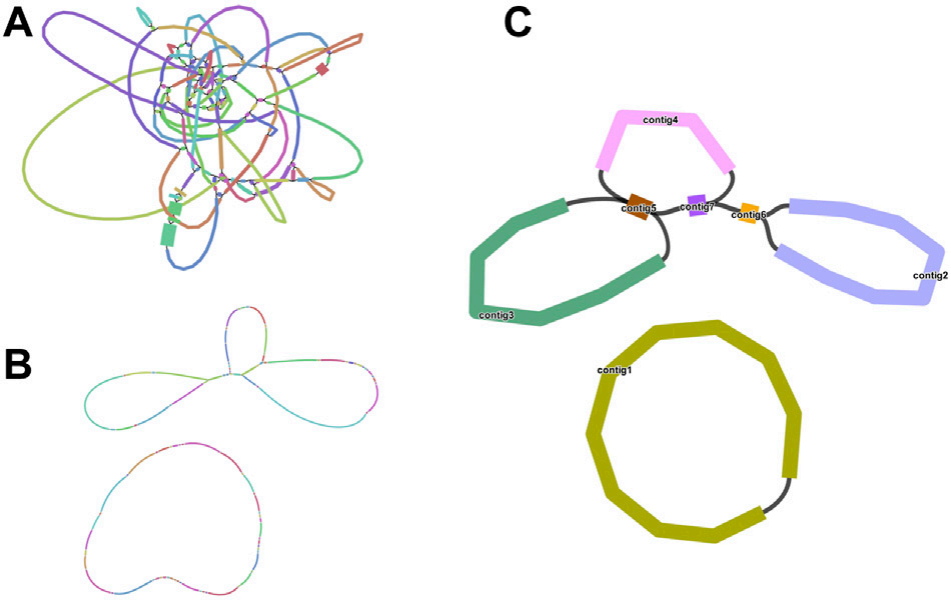
Branched conformation of *C. acerifolia* mitogenome. **(A)**: Sketch of the mitogenome of *C. acerifolia*, containing 136 nodes. **(B)**: The mitogenome sketch obtained after selecting the best path supported by the long read data of ONT and resolving the individual repeat regions. **(C)**: Mitogenome mapping of *C. acerifolia*, with circular DNA molecules comprising contig1 and linear DNA molecules consisting of contig2, contig3, contig4, contig5, contig6 and contig7.

We sequenced DNA samples of *C. acerifolia* using Illumina and Nanopore sequencing platforms to obtain basic data on mitogenome assembly. Among them, Nanopore raw data was 10. 14 Gb, N 50 is 20, 564 bp and Illumina raw data was 13.50 Gb (Supplementary Tables S1, S2). First, we obtained a sketch of the *C. acerifolia* mitogenome assembled from Illumina data, containing a total of 136 nodes that were linked to each other to form overlapping regions, and resolved these repetitive regions in turn by selecting the most appropriate pathway supported by the ONT long read data (Figure 1). This resulted in two independent *C. acerifolia* mitochondrial DNA molecules containing 7 contigs (numbered according to their length), one of which has a closed-loop structure, while the other has a multi-branching structure with a variety of different potential conformations. In order to include all contigs as much as possible, we unfolded them in the order contig3-contig5-contig7-contig4-contig5-contig7-contig6-contig2. There is a dynamic transformation of mitochondrial DNA conformation in plants due to repeat-mediated reasons, so we emphasized that the pathway unraveled here is not unique and that the pathway represents only one of these cases.

**FIGURE 2:**
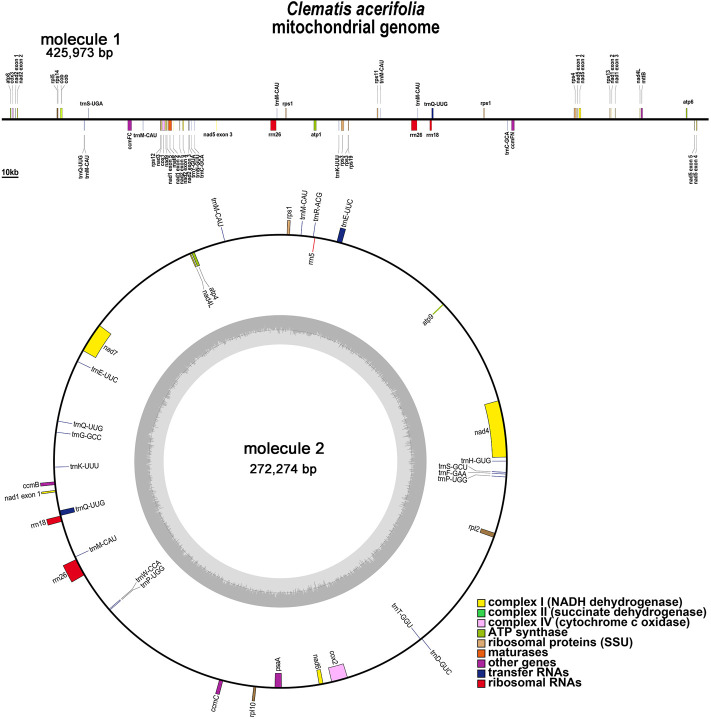
*C. acerifolia* mitogenome gene map. Genes shown on the outside and inside of the circle are transcribed clockwise andcounterclockwise, respectively. The dark gray region in the inner circle depicts GC content.

*C.acerifolia* mitogenome showed a multi-branched structure with a total size of 698, 247 bp and a GC content of 46.91%, consisting of a linear molecule (Molecule1, 425, 973 bp) and a circular molecule (Molecule2, 272, 274 bp) with GC contents of 46.82 and 47.06%, respectively (Figure 2). Fifty-five genes were annotated, including 35 protein-coding genes (PCGs), 17 tRNA genes (*trn*C-GCA, *trn*E-UUC, *trn*K-UUU, *trn*M-CAU, *trn*P-UGG, and *trn*Q-UUG are multi-copy genes), and three rRNA genes (Table 1). The PCGs include 24 unique mitochondrial core and 11 non-core genes. There are seven main categories of core genes, including five ATP synthase genes (*atp*1, *atp*4, *atp*6, *atp*8, and *atp*9); nine NADH dehydrogenase genes (*nad*1, *nad*2, *nad*3, *nad*4, *nad*4L, *nad*5, *nad*6, *nad*7, and *nad*9); four ubiquinol cytochrome c reductase genes (*ccm*B, *ccm*C, *ccm*Fc, and *ccm*Fn); 3 cytochrome C oxidase genes (*cox*1, *cox*2, and *cox*3); 1 maturation enzyme gene (*mat*R); 1 panthenol-cytochrome C reductase gene (*cob*), and 1 membrane transport protein gene (*mtt*B). The non-core genes consist of three ribosomal large subunit genes (*rpl*2, *rpl*5, and *rpl*10) and eight ribosomal small subunit genes (*rps*1, *rps*3, *rps*4, *rps*11, *rps*12, *rps*13, *rps*14, and *rps*19). In Molecule1, *nad*2 and *nad*5 contained 4 introns, *ccm*FC, *rps*3, *rps*4, *rps*11, *trn*Q-UUG contained 1 intron, and there is trans splicing in the *nad*1, *nad*2 and *nad*5 genes, while in Molecule2, *nad*4 and *nad*7 have 3 and 4 introns, respectively, while *cox*2, *trn*E-UUC and *trn*Q-UUG all have one intron.

**TABLE 1:** Gene composition in the *C. acerifolia.*

Group of genes	Name of genes
ATP synthase	*atp1*, *atp4*, *atp6*, *atp8*, *atp9*
NADH dehydrogenase	*nad1*, *nad2*, *nad3*, *nad4*, *nad4L* (×2), *nad5*, *nad6*, *nad7*, *nad9*
Cytochrome c biogenesis	*cob* (×2)
Ubiquinol cytochrome c reductase	*ccmB*, *ccmC*, *ccmFC*, *ccmFN*
Cytochrome c oxidase	*cox1*, *cox2*, *cox3*
Maturases	*matR*
Transport membrane protein	*mttB*
Large subunit of ribosome	*rpl10*, *rpl2*, *rpl5*
Small subunit of ribosome	*rps1* (×3)*, rps11*, *rps12*, *rps13*, *rps14*, *rps19*, *rps3*, *rps4*
Ribosome RNA	*rrn18* (×2), *rrn26* (×3), *rrn5*
Transfer RNA	*trnC*-*GCA* (×2), *trnD*-*GUC*, *trnE*-*UUC* (×2), *trnF*-*GAA*, *trnG*-*GCC*, *trnH*-*GUG*, *trnK*-*UUU* (×2), *trnM*-*CAU* (×8), *trnN*-*GUU*, *trnP*-*UGG* (×2), *trnQ*-*UUG* (×4), *trnR*-*ACG*, *trnS*-*GCU*, *trnS*-*UAG*, *trnT*-*GGU*, *trn*W-CCA, *trn*Y-GUA

### PCGs codon usage preference and RNA editing sites prediction

**FIGURE 3:**
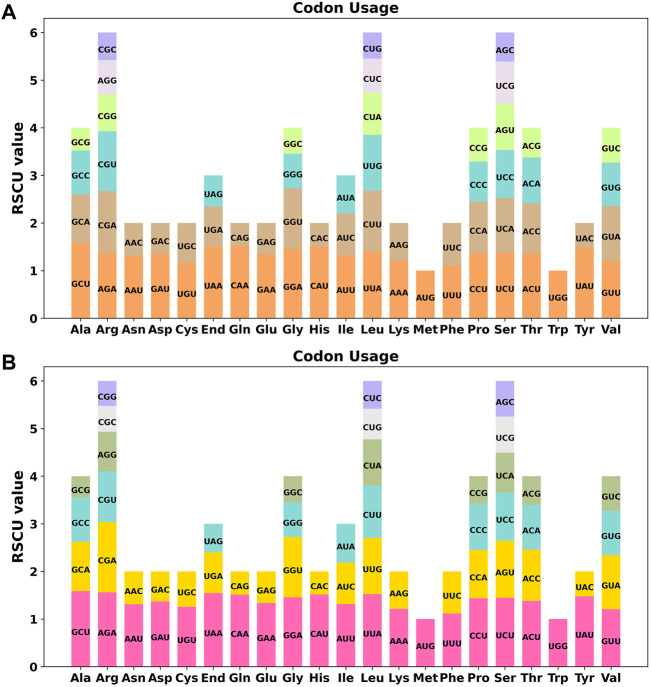
*C. acerifolia* mitogenome relative synonymous codon usage (RSCU). **(A)** Before RNA editing. **(B)** After RNA editing. Codon families are shown on the x-axis. RSCU values are the number of times a particular codon is observed relative to the number of times that codon would be expected for a uniform synonymous codon usage.

Codon usage preference is usually considered to be the result of the tendency to develop relative equilibrium within cells during species evolution, and codons with a relative synonymous codon usage (RSCU) greater than 1 are considered to be used by amino acid preference. As shown in Figure 3A, the codon preference analysis of 35 PCGs revealed that GCU (Ala), UAA (End), CAA (Gln), CAU (His), and UAU (Tyr) were the most common codons, with Ala having the highest RSCU value of 1.58 for the GCU codon in mitochondrial PCGs (Table S3). There were 26 codons with RSCU values greater than 1. Except for two codons, AUG and UGG, other codons ending in A/U (T) bases accounted for 92.3%. Except for the methionine (AUG) and tryptophan (UGG) start codons with RSCU values of 1, there is a broad codon usage preference for PCGs in *C. acerifolia* mitogenome.

Neutrality plot analysis allows preliminary determination of the factors affecting codon preference. Supplementary Figure S1A shows that GC12 was between 0.3462–0.5254 and GC3 was between 0.2533–0.5799. Codon GC3 was significantly higher than GC12 for most genes, and GC12 and GC3 correlation analysis was significantly correlated (Supplementary Table S4), with most genes near the diagonal but some genes slightly further away from the diagonal, indicating that *C. acerifolia* mitogenome codon usage preference, although influenced by natural selection, is more influenced by gene mutations. ENC-plot can further determine the factors affecting codon preference. Most of the gene sites are distributed near the standard curve (Supplementary Figure S1B), which means that the effect of mutational pressure plays a dominant role in the formation of codon preference.

**FIGURE 4:**
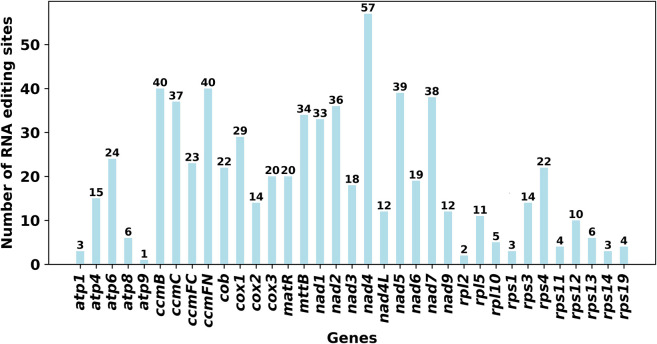
Number of RNA editing sites predicted by individual PCGs in mitochondria.

We used PREP (predictive RNA editors for plants) suit to identify RNA edits for the 35 PCGs in the *C. acerifolia* mitogenome (Figure 4). A total of 676 potential RNA editing sites were identified for the 35 PCGs at a cutoff value = 0.2 criterion, and all were clip C to U edits. *Nad*4 gene identified 57 RNA editing sites, the highest number of edits among all mitochondrial genes. This was followed by the *ccm*FN and *ccm*B genes, each had 40 RNA editing events. The *atp*9 gene was predicted to have only one RNA editing event, the least of all genes. The comparative analysis of codon usage before and after RNA editing showed that the preference of most codons did not change (Figure 3B and Supplementary Table S3), but we also noticed that some amino acids such as AGA (Arg), CGA (Arg), UAA (End), CCU (Pro), CCC (Pro) and UCU (Ser) were significantly enhanced after the RNA editing event.

### Repeats analysis in *C. acerifolia* mitogenome

**FIGURE 5:**
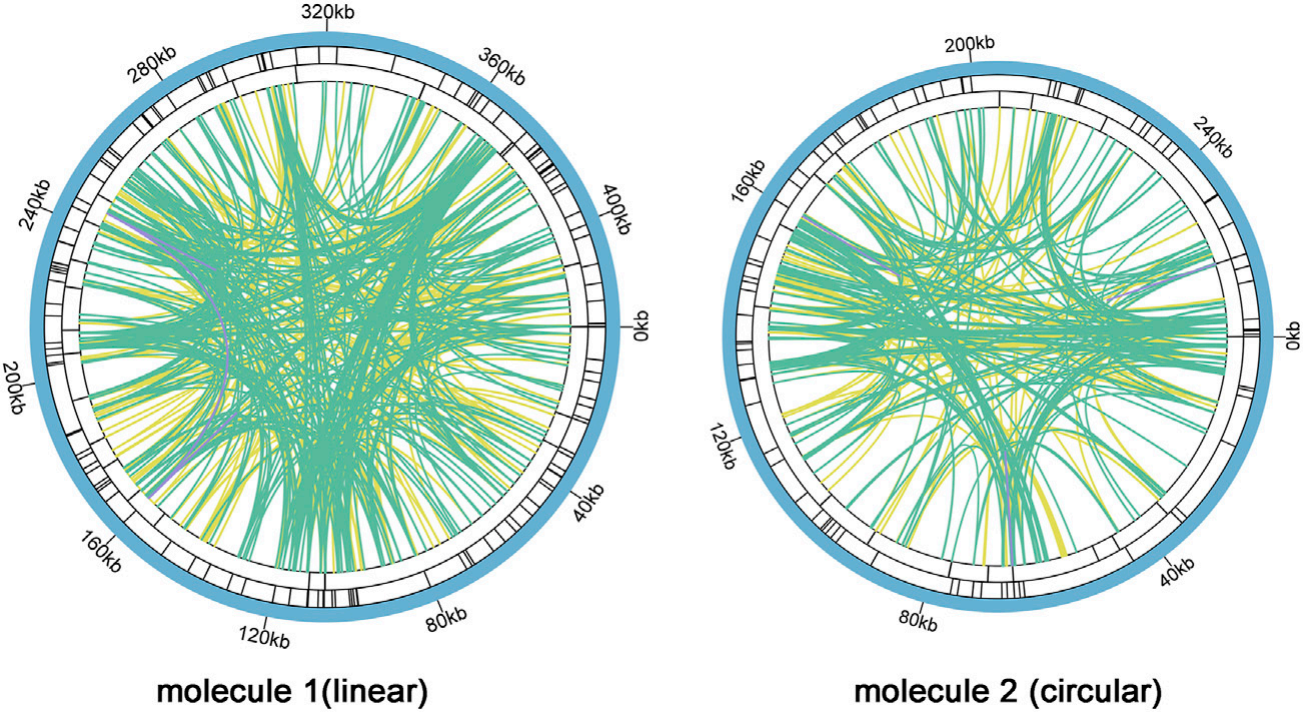
*C. acerifolia* mitogenome repeatedsequence diagram. Two mitochondrial molecules were analyzed separately forrepetitive sequences (one linear and one circular), taking the linear molecule 1 as an example. For example, the colored lines on the innermost circle connect the two repetitive sequences of TEs, the green line represents Palindromic Match (P), the yellow line represents Forward Match (F), and the purple line represents Reverse Match (R). The black line on the second circle represents TRs, and the black line on the outermost circle represents SSRs. molecule 2 and so on.

There are a large number of repeats in biological cell DNA sequences including tandem repeats (TRs) and interspersed repeats (TEs), which can be classified by size as large (LR, >500 bp), medium (IntR, 50–500 bp), and short (SR, <50 bp) repeats (Gualberto and Newton, 2017). Microsatellite repeats are a special kind of presence among TRs, which can better reflect species’ genetic structure and genetic diversity changes (Li et al., 2019). We found 126 and 81 SSRs in molecule 1 (linear) and molecule 2 (circular), respectively (Figure 5, SupplementaryTable S7 and Supplementary Table S10). In molecule 1 (linear), most SSRs were in tetrameric form, accounting for 32.54% (41) of the total SSRs, with AAGA and GAAG being the most common types of tetramers. The least number of hexamers (3); among the 28 monomeric SSRs, adenine (A) monomeric repeats were the most abundant (15), accounting for 53.57% of the monomeric SSRs. The tetrameric form of SSRs was also the most abundant in molecule 2 (circular), accounting for 37.04% (30) of the total SSRs, with two of each of the four types CATT, CAAG, TAGA, and AAGG in the tetramer and only one of the other types of tetramers. In contrast the pentamer was the least with only one. SSRs in monomeric and dimeric forms accounted for 44.44% of the total SSRs. Thymine (T) monomeric repeats accounted for 56.25% (9) of the 16 monomeric SSRs. Among the two mitochondrial molecules, TA repeat sequences were the most common type of dimeric SSRs, accounting for 30.77 and 25.00% of the dimeric SSRs, respectively.

Analysis of TRs and TEs of the mitogenome revealed 24 TRs with greater than 61% match and length between 9 and 50 bp in M1 (Supplementary Table S5); 680 pairs of TEs with length greater than or equal to 30, including 335 pairs of Forward Match, 342 pairs of Palindromic Match and 3 pairs of Reverse Match, and no Complement Match detected (Supplementary Table S6). The longest Forward Match was 11,827 bp. A total of 16 TRs in M2 with 81% or more match and length between 3 and 39 bp (Supplementary Table S8); 447 pairs of TEs with length greater than or equal to 30, including 201 pairs of Forward Match, 241 pairs of Palindromic Match 241 pairs, Reverse Match 3 pairs, and Complement Match 2 pairs were deteected (Supplementary Table S9). The longest Forward Match is 243 bp.

### Intracellular gene transfer of *C. acerifolia* organelle genomes

**FIGURE 6:**
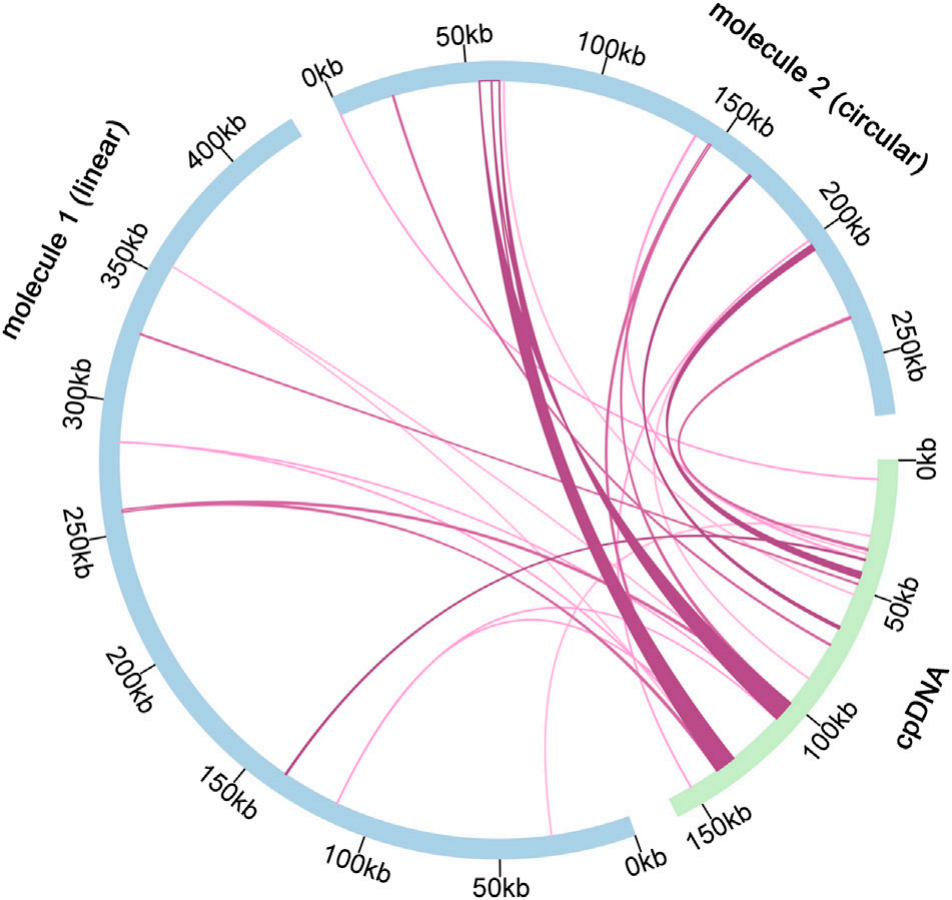
Schematic representation of gene transfers between chloroplast and mitogenome in *C. acerifolia*. The blue arcs in the diagram represent the mitogenome, the green arcs represent the chloroplast genome, and the rosy red lines between the arcs correspond to the genomic fragments that are homologous.

According to sequence similarity analysis (Figure 6 and Supplementary Table S11), a total of 28 fragments were homologous to the mitochondrial and chloroplast genomes, with a total length of 25, 049 bp. In addition, five of the 28 homologous fragments exceeded 1, 000 bp, with fragments 12 and 13 being the longest at 4, 643 bp. By annotation of these homologous sequences, 13 complete genes were also found on 28 homologous fragments, including 2 PCGs (*pet*L and *pet*G), 9 tRNA genes (*trn*N-GUU, *trn*A-UGC, *trn*I-GAU, *trn*N-GUU, *trn*A-UGC, *trn*I-GAU, *trn*N-GUU, *trn*A-UGC, *trn*I-GAU, and *trn*N-GAU). *Trn*R-ACG, *trn*W-CCA, *trn*P-UGG, *trn*D-GUC, *trn*H-GUG, *trn*M-CAU) and two rRNA genes (*rrn*5S and *rrn*4.5 S).

### Phylogenetic inference and synteny analysis

**FIGURE 7:**
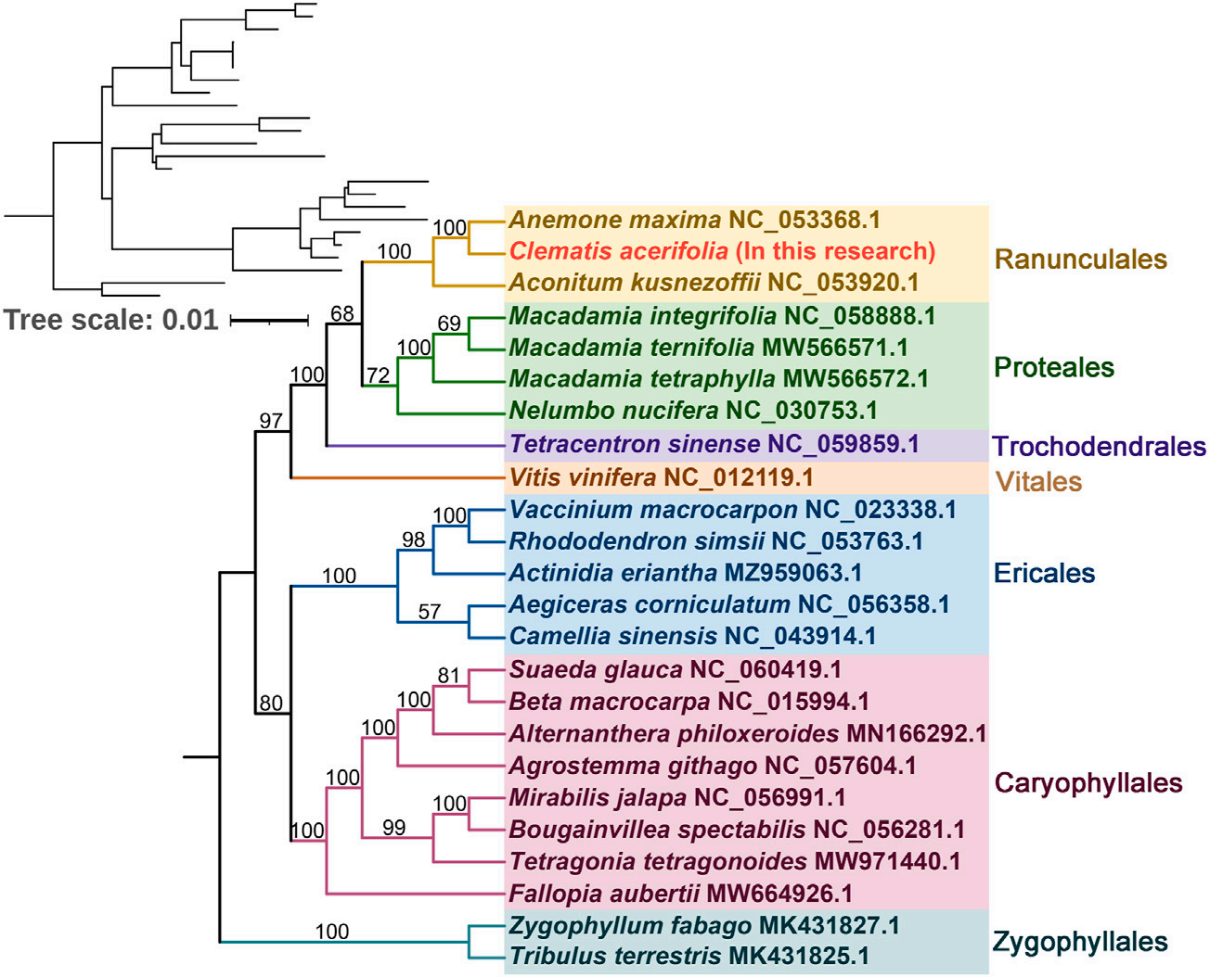
Phylogenetic tree of 24 angiosperms based on the sequences of 39 conserved mitochondrial PCGs. *Zygophyllum fabago* and *Tribulus terrestris* were selected as outgroups. The number at each node is the bootstrap probability.

**FIGURE 8:**
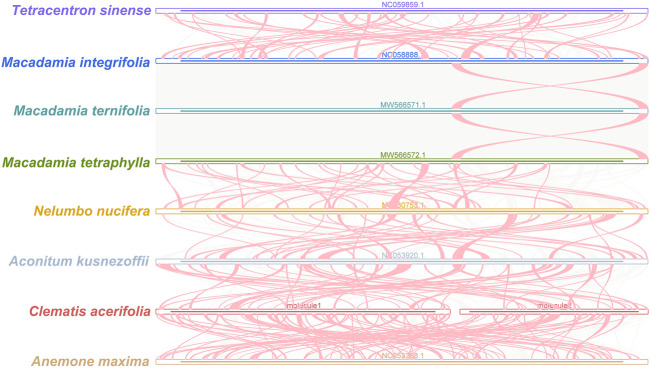
*C. acerifolia* mitogenome synteny. Bars indicated the mitogenomes, and the ribbons showed the homologous sequences between the adjacent species. The red areas indicate where the inversion occurred, the gray areas indicate regions of good homology. Common blocks less than 0.5 kb in length are not retained, and regions that fail to have a common block indicate thatthey are unique to the species.

To understand the evolutionary status of *C. acerifolia*, based on 39 conserved mitochondrial PCGs for seven orders of angiosperms (Ranunculales, Proteales, Trochodendrales, Vitales, Ericales, Caryophyllales, and Zygophyllales), were constructed as phylogenetic trees, and *Zygophyllum fabago* and *Tribulus terrestris* of the Zygophyllales were set as outgroups (see Supplementary Table S12, for mitogenome abbreviations and accession numbers). As shown Figure 7 Figure 8, all seven taxa of the studied order showed good clustering, and this phylogenetic tree strongly supports the clustering of *C. acerifolia*, *A. kusnezoffii* and *A. maxima* into one group, with *C. acerifolia* being more closely related to *A. maxima* and belonging to the Ranunculaceae of Ranunculales.

**FIGURE 9:**
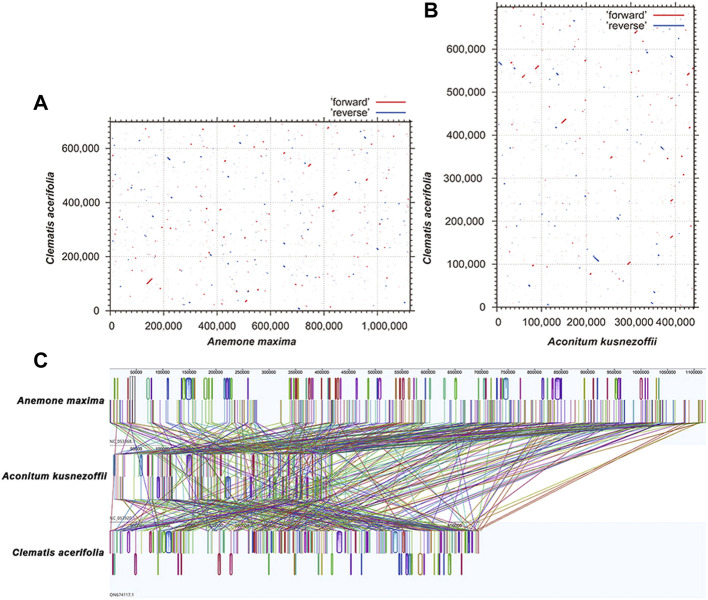
Dot plot and collinearity analysis of the mitogenomes of *C. acerifolia*, *A. maxima* and *A. kusnezoffii*. **(A, B)** are dot plots of *A. maxima* and *A. kusnezoffii* with *C. acerifolia*, respectively. **(C)** is the collinearity analysis between mitogenomes, with homologous regions represented by the same color blocks and connected by lines.

Plant mitogenomes are extremely conserved in terms of the number and types of functional genes as well as their sequences, but functional genes vary greatly in the location and order of mitogenomes of different species (Tan and Pang, 2013). By comparing the genome sequences of some closely related species, large co-linear blocks can be obtained, and these regions include very rich homology information, so the degree of co-linearity can be used to measure the kinship and evolutionary distance between species, as well as to discover unknown genes and improve the quality of genome annotation (McCouch, 2001). Based on sequence similarity, we mapped Multiple Synteny Plot of *C. acerifolia* with seven closely related species. A large number of homologous co-linear blocks were detected between *C. acerifolia* and its closely related species, especially with *A. maxima* of *Anemone*. The dot plot analysis showed that there were only sporadic collinear regions between the three mitogenomes of *C. acerifolia*, *A. maxima* and *A. kusnezoffii*, showing poor collinearity (Figures 9A,B). Similar results were obtained by Mauve collinearity analysis (Figure 9C). We found that the co-linear blocks were not arranged in the same order between different mitogenomes, which means that the *C. acerifolia* mitogenome has undergone extensive genomic rearrangements, and the *C. acerifolia* mitogenome is extremely unconserved in structure. In addition, it is particularly noteworthy that the three *Macadamia* species (*Macadamia integrifolia*, *Macadamia ternifolia*, and *Macadamia tetraphylla*) share extremely high sequence homology with each other and no rearrangement events were detected.

## Discussion

### Size and structure of the mitochondrial genome

Plant mitogenomes size are much larger than other species. For example, animal mitogenomes are usually only 15–17 kb, algal mitochondria are slightly larger and can reach 13–96 kb, while angiosperm mitogenome size is usually in the range of 200–700 kb (Gualberto and Newton, 2017), *Silene conica* (11.3 Mb) is the largest plant mitogenome found so far (Sloan et al., 2012). *C. acerifolia* mitogenome size is between that of *A. kusnezoffii* (440, 720bp) and *A. maxima* (1, 122, 546) of the Ranunculaceae (Park and Park, 2020; Li et al., 2021). Although plant mitogenomes are very large, they do not contain many coding genes, typically 50–60 (Kitazaki and Kubo, 2010). With 55 genes (35 PCGs) annotated in the *C. acerifolia* mitogenome, which is close to the number of genes annotated in *Actinidia chinensis* (Wang et al., 2019), *Quercus acutissima* (Liu et al., 2022), and *Populus simonii* (Bi et al., 2022)*.* The sequences of the coding regions of angiosperms account for only 7–17% of the total genome, and the rest is occupied by intergenic regions (Kubo and Newton, 2008).

GC content is also one of the important indicators for species evaluation, and *C. acerifolia* mitogenome GC content was 46.91%, which is similar to the GC content of the mitogenomes of most angiosperms (*Q. acutissima*, 45.72%; *Dalbergia odorifera*, 45.1%, *Cucumis hystrix*, 44.59%; *Elymus sibiricus*; 44.47%) and higher than that of the *clematis* chloroplast genome (37.87–38.19%) (Hong et al., 2021; Liu et al., 2022; Xia et al., 2022). The *C. acerifolia* mitogenome exhibits a multi-branched structure (linear molecule 1 and circular molecule 2), and the vast majority of published plant mitogenomes are presented as circular DNA molecules, and there are also conformational forms such as Y- and H-type linear, but mitochondrial DNA in its real state is often a mixture of different conformational forms (Backert and Börner, 2000; Kitazaki and Kubo, 2010; Bonen, 2014; Christensen, 2019).

### Codon usage preferences and RNA editing sites in organelle genomic PCGs

Codon usage preference analysis provides a better understanding of the role of the mitogenome in evolution. Although the mitogenomes of different species may differ in size, structure, and sequence, there is a great deal of similarity in the gene products they encode (Li et al., 2011). Analysis of the codon usage preferences of *C. acerifolia* mitogenome reveals that, like most other plants, Leu, Ser, and Arg are the most common amino acids, compared to Met and Trp, which are much less common (Ma et al., 2022). The preference of genes encoded in *C. acerifolia* mitogenome for codons ending in A/T is consistent with the codon preferences of *A. thaliana* and *Nicotiana tabacum* (Duret and Mouchiroud, 1999), in contrast to monocotyledons (G/C base endings) (Mazumdar et al., 2017). An increasing number of studies have shown that genetic codon usage preference is an important feature of biological evolution, but there is no single influencing factor responsible for this feature; in addition to the effect of mutational pressure (Rao et al., 2011), natural selection (Wang et al., 2022), tRNA abundance (Behura and Severson, 2010), gene length (Wei et al., 2014), and expression level (Benjamini and Speed, 2012) all contribute to codon usage preferences. In this study, codon usage preference in the *C. acerifolia* mitogenome was influenced by both mutational and selective pressures, with mutational pressure playing a dominant role, but the exact mechanism of influence needs to be further investigated.

RNA editing is one of the required steps for gene expression in plant mitogenomes and is widespread in higher plant mitochondria, occurring mainly at one of the first two positions of the codon, which affects its amino acid alterations and plays a crucial role in plant evolution (Edera et al., 2018). Some amino acids with good codon usage preference in the mitogenome of *C. acerifolia* are significantly enhanced after RNA editing. The occurrence frequency of editing sites varies widely among plants, with more than 2,000 sites having been reported in bryophytes (Hecht et al., 2011), between 200–700 in angiosperms (Mower, 2009; Sloan et al., 2010; Richardson et al., 2013), and about 500 in gymnosperms (Salmans et al., 2010), while some plants, such as green algae, have not been found to have RNA editing sites. Unlike other plants, angiosperms are experiencing a loss of editing sites at this stage, mainly due to the replacement of editable thymidine by cytosine in the genome (Shields and Wolfe, 1997; Cuenca et al., 2010). In *C. acerifolia*, 676 RNA editing sites were identified, similar to the number of editing sites in the Ranunculaceae plant *A. maxima* (687) (Park and Park, 2020). RNA editing includes different forms such as C to U, U to C, and A to I (Small et al., 2020). However, all of the *C. acerifolia* mitochondria genomes were edited by editing C to U. Notably, the stop codon of the plant mitochondrial atp9 gene, which was generated by RNA editing in 60% of the plants, was predicted only once in *C. acerifolia* (Handa, 1993). In addition, abnormal RNA editing in organelles affecting embryonic endosperm development, plant growth and abiotic stress resistance has been elaborated on in many studies, which also indicates that RNA editing has a non-negligible role in plant growth and development (Yan et al., 2018).

### Identification of repeat sequences

Containing abundant repeats is one of the distinctive features of mitogenomes in higher plants. The large number of repeats leads to frequent homologous recombination and is more prone to gene rearrangement events, which is closely related to the complexity of mitochondrial gene structure (Stern and Palmer, 1984).

TRs and TEs play an important role in genome evolution, with high mutation rates of TRs accelerating gene coding and regulatory sequence evolution (Gemayel et al., 2010), and TEs being able to alter DNA sequences through a series of events such as cleavage, duplication, and reintegration (Han, 2012). It has been shown that these repeats are active in plant mitogenome reorganization, affecting structural changes in the mitogenome and producing greater mitogenome size (Guo et al., 2016). There are three pairs of LRs in *C. acerifolia* mitogenome, with the longest TEs exceeding 10 kb (11, 827 bp), and we speculate that intra- and inter-molecular recombination may have occurred through long repeats, leading to the generation of heteromeric forms or subgenomic molecules in the *C. acerifolia* mitogenome.

### Intracellular gene transfer of *C. acerifolia* organelle genomes

We mentioned earlier, non-coding sequences constitute a large part of plant mitogenomes, consisting of repeat fragments, sequences transferred from the chloroplast and nuclear genomes, and even sequences transferred at the gene level to other species. Gene fragments from chloroplasts are commonly present in the mitogenomes of higher plants and represent a high proportion of them (Zhang et al., 2011). The mitogenome of *Amborella trichopoda*, the oldest angiosperm, contains a large number of sequence fragments from mosses, green algae and other angiosperms (Rice et al., 2013).

Length of homologous fragments between *C. acerifolia* mitogenome and chloroplast genome accounted for 3.59% of the total mitogenome, while the mitogenome of *Vitis vinifera* contained 30 fragments from the chloroplast, totaling 68, 237 bp, accounting for 8.8% of the entire mitogenome and 42.4% of its entire chloroplast genome, this value is the largest among sequenced plant mitogenomes (Goremykin et al., 2009). Interestingly, we found that the proportion of chloroplast DNA sequences was much lower in *C. acerifolia*, but compared with the lower plant *Marchantia polymorpha*, which has 27 tRNAs but no chloroplast-derived sequences (Oda et al., 1992), the transfer of chloroplast gene sequences to mitochondria may be unique to flowering plants.

### Phylogenetic inference and synteny analysis

In our study, *C. acerifolia*, *A. maxima*, and *A. kusnezoffii* were grouped together in the Ranunculaceae, and the topology of the mitochondrial DNA-based phylogeny coincided with the Angiosperm Phylogeny Group (APG) classification. The order of the 24 species in the phylogenetic tree is consistent with the species evolutionary relationships, which also illustrates the consistency between traditional and molecular taxonomy. Previously, *C. acerifolia* was traditionally classified in the subsection *Montanae Schneider* (Grey-Wilson, 2000; Johnson, 2001), while Wang’s recent revision of the genus *Clematis* placed it in Sect. *Cheiropsis* as a single species in Subsect (Wang, 2002; Wang and Li, 2005). Xie (2011) also considered *C. acerifolia* to be a phylogenetically isolated lineage and speculated that this species may represent a relic species of Holarctic *Clematis*.

The co-linearity between *C. acerifolia* mitogenomic and *A. maxima* is high, with large homozygous co-linear blocks, suggesting that the two species have undergone extensive rearrangement phenomena. We speculate that a break fusion occurred between their different chromosomes, that is, *C. acerifolia* and *A. maxima* did not diverge simultaneously, but rather as one species evolved from the other. Wang’s analysis of important morphological characters, such as plant trophic and reproductive organs, revealed that *Clematis* is adjacent to *Trollius* and *Anemone* and originated from *Anemone* (Wang and Li, 2005). Tamura also highlighted the existence of a close kinship between *Clematis* and *Anemone* (Tamura, 1955; Tamura, 1967; Tamura, 1970; Tamura, 1987; Tamura, 1995; Tamura, 1997). All these studies confirm the accuracy of the mitogenome of *C. acerifolia* in determining its phylogenetic position.

## Conclusion

*Clematis* is one of the most widespread genera of flowering plants, and its ornamental value and species diversity have evoked research and exploration. In recent years, *C. acerifolia* has attracted much attention, but the phylogenetic status of the genus is highly controversial. Here, we have assembled and annotated the complete mitogenome of *C. acerifolia*, the first mitogenome of *Clematis*. The *C. acerifolia* mitogenome is a relatively rare multi-branched structure that including a linear molecule (M1) and a circular molecule (M2), which we speculate may have some association with long repeats. We performed extensive analyses based on the DNA and amino acid sequences of the annotated genes, and we found that mutational pressure was the main factor influencing codon usage preferences. In addition, *C. acerifolia* has similar affinities to *A. maxima* and substantial genomic rearrangements occur between it and closely related species. Our study is expected to enrich the information on the organelle genome of *C. acerifolia* and provide many new insights into *Clematis* genetics and evolution, but the determination of the position of *C. acerifolia* in the genus still requires the addition of more genetic information, and we hope that subsequent studies will help better clarification of these relationships.

## Data Availability

The datasets presented in this study can be found in online repositories. The names of the repository/repositories and accession number(s) can be found in the article/Supplementary Material.
