# Supplementary material for: Complete sequence and comparative analysis of the mitochondrial genome of the rare and endangered Clematis acerifolia, the first clematis mitogenome to provide new insights into the phylogenetic evolutionary status of the genus
Source: Front Genet. 2023 Jan 4;13:1050040. doi: 10.3389/fgene.2022.1050040 (PMC9907779; doi:10.3389/fgene.2022.1050040)
Supplement: Supplementary file 3 [file Table1.docx]

Supplementary Material

**Table S1** Statistics of Illumina sequencing data.

| **Items** |  |
| --- | --- |
| Length(bp) | 150; 150 |
| Q20(%) | 98. 38; 98. 61 |
| Q30(%) | 94. 57; 94. 66 |
| GC Content(%) | 38. 89; 38. 85 |
| Total Reads | 9, 0009, 870 |
| Total Bases | 13, 501, 480, 500 |
| Note: Length is the length of reads; Q20 is the proportion of nucleotides with quality value larger than 20; Q30 is the proportion of nucleotides with quality value larger than 30; GC Content is the proportion of bases G and C; Total Reads is total number of raw reads; Total Bases is total nucleotides number of raw reads. | |

**Table S2** Statistics of Nanopore sequencing data.

| **Items** | **All(bp)** | **Pass(bp)** |
| --- | --- | --- |
| Total Bases | 11, 186, 623, 578 | 10, 143, 619, 495 |
| Total Reads | 1, 196, 898 | 1, 081, 110 |
| MaxLen | 170, 546 | 170, 546 |
| AvgLen | 9, 346. 34 | 9, 382. 59 |
| N50 | 20, 494 | 20, 564 |
| L50 | 170, 309 | 154, 271 |
| N90 | 4, 492 | 4, 516 |
| L90 | 604, 604 | 546, 061 |
| MeanQ | 9. 92 | 10. 33 |
| Note: All is all sequencing data, Pass is valid sequencing data; Total Bases is the number of bases; Total Reads is the number of reads; MaxLen is the maximum length of the data; AvgLen is the average length of the data; N50 (N90) is the length of the last added read when the summed length reaches 50% (90%) of the total length of all the reads after sequentially accumulating all the reads in order from longest to shortest; L50 (L90) is the total number of sequential entries when the summed length reaches 50% (90%) of the total length of all the reads after sequentially accumulating all the reads in order from longest to shortest; MeanQ is the average quality value. | | |

Table S3Comparison of RSCU values of amino acids in *C. acerifolia* mitogenome before and after RNA editing.

| **Amino** | **codon 1**  **RSCU** | **codon 2**  **RSCU** | **codon 3**  **RSCU** | **codon 4**  **RSCU** | **codon 5**  **RSCU** | **codon 6**  **RSCU** |
| --- | --- | --- | --- | --- | --- | --- |
| Ala | GCU | GCA | GCC | GCG |  |  |
| after RNA editing | 1.59 | 1.03 | 0.93 | 0.45 |  |  |
| before RNA editing | 1.58 | 1.02 | 0.92 | 0.49 |  |  |
| Arg | AGA | CGA | CGU | AGG | CGC | CGG |
| after RNA editing | 1.56 | 1.48 | 1.07 | 0.82 | 0.54 | 0.53 |
| before RNA editing | 1.36 | 1.29 | 1.25 | 0.78 | 0.73 | 0.59 |
| Asn | AAU | AAC |  |  |  |  |
| after RNA editing | 1.31 | 0.69 |  |  |  |  |
| before RNA editing | 1.31 | 0.69 |  |  |  |  |
| Asp | GAU | GAC |  |  |  |  |
| after RNA editing | 1.37 | 0.63 |  |  |  |  |
| before RNA editing | 1.37 | 0.63 |  |  |  |  |
| Cys | UGU | UGC |  |  |  |  |
| after RNA editing | 1.26 | 0.74 |  |  |  |  |
| before RNA editing | 1.16 | 0.84 |  |  |  |  |
| End (Stop codon) | UAA | UGA | UAG |  |  |  |
| after RNA editing | 1.54 | 0.86 | 0.60 |  |  |  |
| before RNA editing | 1.54 | 0.86 | 0.60 |  |  |  |
| Gln | CAA | CAG |  |  |  |  |
| after RNA editing | 1.51 | 0.49 |  |  |  |  |
| before RNA editing | 1.52 | 0.48 |  |  |  |  |
| Glu | GAA | GAG |  |  |  |  |
| after RNA editing | 1.34 | 0.66 |  |  |  |  |
| before RNA editing | 1.34 | 0.66 |  |  |  |  |
| Gly | GGA | GGU | GGG | GGC |  |  |
| after RNA editing | 1.46 | 1.27 | 0.73 | 0.55 |  |  |
| before RNA editing | 1.46 | 1.27 | 0.73 | 0.55 |  |  |
| His | CAU | CAC |  |  |  |  |
| after RNA editing | 1.51 | 0.49 |  |  |  |  |
| before RNA editing | 1.50 | 0.50 |  |  |  |  |
| Ile | AUU | AUC | AUA |  |  |  |
| after RNA editing | 1.31 | 0.87 | 0.82 |  |  |  |
| before RNA editing | 1.32 | 0.87 | 0.81 |  |  |  |
| Leu | UUA | UUG | CUU | CUA | CUG | CUC |
| after RNA editing | 1.52 | 1.18 | 1.10 | 0.97 | 0.64 | 0.58 |
| before RNA editing | 1.39 | 1.29 | 1.16 | 0.89 | 0.71 | 0.56 |
| Lys | AAA | AAG |  |  |  |  |
| after RNA editing | 1.21 | 0.79 |  |  |  |  |
| before RNA editing | 1.21 | 0.79 |  |  |  |  |
| Met | AUG |  |  |  |  |  |
| after RNA editing | 1.00 |  |  |  |  |  |
| before RNA editing | 1.00 |  |  |  |  |  |
| Phe | UUU | UUC |  |  |  |  |
| after RNA editing | 1.11 | 0.89 |  |  |  |  |
| before RNA editing | 1.10 | 0.89 |  |  |  |  |
| Pro | CCU | CCA | CCC | CCG |  |  |
| after RNA editing | 1.43 | 1.01 | 0.98 | 0.57 |  |  |
| before RNA editing | 1.36 | 1.10 | 0.84 | 0.71 |  |  |
| Ser | UCU | AGU | UCC | UCA | UCG | AGC |
| after RNA editing | 1.45 | 1.20 | 1.02 | 0.84 | 0.76 | 0.75 |
| before RNA editing | 1.36 | 1.14 | 1.01 | 0.99 | 0.88 | 0.62 |
| Thr | ACU | ACC | ACA | ACG |  |  |
| after RNA editing | 1.38 | 1.08 | 0.94 | 0.60 |  |  |
| before RNA editing | 1.37 | 1.05 | 0.95 | 0.63 |  |  |
| Trp | UGG |  |  |  |  |  |
| after RNA editing | 1.00 |  |  |  |  |  |
| before RNA editing | 1.00 |  |  |  |  |  |
| Tyr | UAU | UAC |  |  |  |  |
| after RNA editing | 1.47 | 0.53 |  |  |  |  |
| before RNA editing | 1.49 | 0.51 |  |  |  |  |
| Val | GUU | GUA | GUG | GUC |  |  |
| after RNA editing | 1.20 | 1.14 | 0.94 | 0.72 |  |  |
| before RNA editing | 1.21 | 1.15 | 0.92 | 0.73 |  |  |

**Table S4** Correlation analysis of codon preference parameters of the mitogenome of *C. acerifolia.*

|  | **GC_all_** | **GC_1_** | **GC_2_** | **GC_3_** | **GC_12_** | **ENC** |
| --- | --- | --- | --- | --- | --- | --- |
| **GC_all_** | 1 |  |  |  |  |  |
| **GC_1_** | 0.488^**^ | 1 |  |  |  |  |
| **GC_2_** | -0.155 | -0.546^**^ | 1 |  |  |  |
| **GC_3_** | -0.317^**^ | -0.589^**^ | -0.197 | 1 |  |  |
| **GC_12_** | 0.23 | -0.331^**^ | 0.344^**^ | 0.466^**^ | 1 |  |
| **ENC** | 0.752^**^ | 0.501^**^ | 0.766^**^ | 0.764^**^ | 0.688^**^ | 1 |
| Note: GC_all_ is the mean GC content of each gene; ^**^Significantly correlated (P<0.05). | | | | | | |


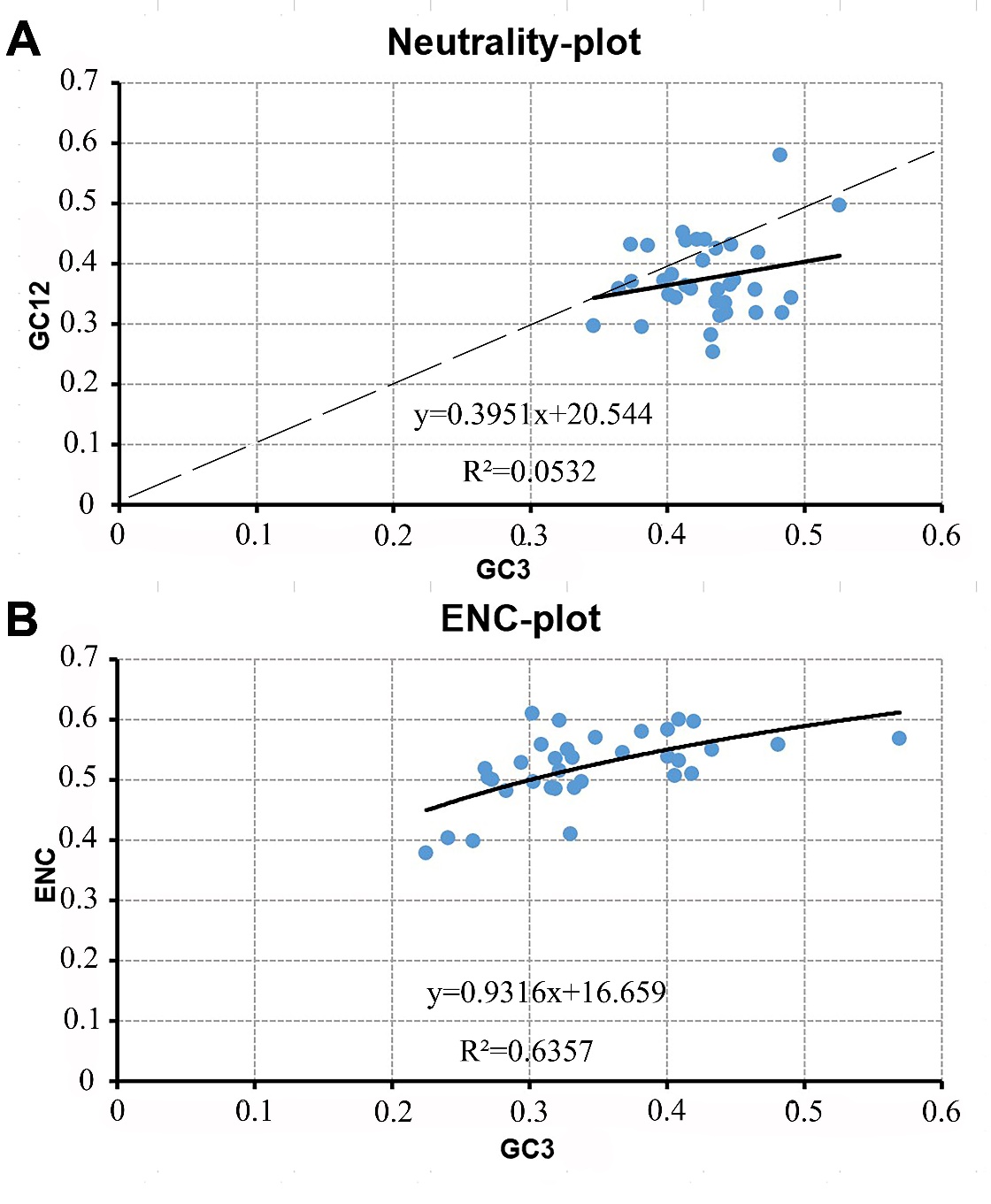


**Figure S1.**

Neutrality plot analysis and ENC-plot analysis for the 35 mitochondrial genes. **A.** Neutrality plot. If the scatter points are all on the diagonal, it means that codon preference is influenced by mutational pressure, otherwise it is influenced by natural selection. **B.** ENC-plot.
